# Concerning the Apprentice System

**Published:** 1883-01

**Authors:** Ben. H. Pratt


### THE BISTOURY.

Vol. ~~XIX.~~ XVIII ELMIRA, N. Y., JANUARY, 1883. No. 4.

### Contributions.

### CONCERNING THE APPRENTICE SYSTEM.

BEN. H. PRATT.

Unions, among craftsmen of every ilk, exist. Trades unions are not new, but late years have developed therein some features which are not the result of wisdom. Doubtless united action is quite necessary on the part of men engaged in any of the established vocations, especially those pursued by artisans in the employ of capitalists. The commonly received idea is that such union is solely protective. Without question, nine-tenths of those who to-day are members of unions of the several sorts, became such simply to provide against the possible advantage which unprincipled masters may be inclined to take of those whom they employ. On the whole, such combination of labor has doubtless inured to the general good of those who maintain a livelihood by the sweat of the brow and the dexterity of the hand, and the strength of the muscle. As these unions exist to-day, they are not regarded as designed with any defiant or menacing purpose. The honorable master recognizes and respects them; the master without such instinct respects and fears them. "In union there is strength" is not so favorite a motto with the latter day men of trades unions, as this: "In a multitude of counsel there is wisdom." These combinations are not the impulsive hordes of ignorant men that they used to be. The peaceful, non-combatant, argumentative, compromising, conciliatory, arbitrational elements of trades unions have largely developed during the past decade, and these features have won not only the self-respect of the membership of the unions, but the admiration of those against whom unions are the avowed protectors and guards. Masters are no longer intolerant of these organizations, and they are no longer conducted in secret. Every manufacturer, mine operator, merchant, builder or other employer of men, no longer eyes askance a union man. It is taken for granted that every guild has its protective organization, and that every good workman is a member thereof. It is no longer an objection to an applicant for place that he is a member of a union consisting of men of his trade or calling or profession, but there is a suspicion lurking about the man who does not belong to such an organization. The question is more frequently put, in the case of an application for work: "are you in good standing in your union?" than "are you a member of the union of your craft?" It has come to pass, therefore, that the very thing which aforetime was a ground of objection and suspicion, and a frequent cause of a denial of employment, has become a recommendation and a reason for the acceptance of service. All of this proves that trades unions are not bad institutions; that controlled as they are, by moderation, and governed by the highest wisdom of its constituency, they are not only elevators of the moral standard of the working classes, but are the educators of the craftsmanship of every toiler. In a word, we have better mechanics, better servitors of every class and condition of men, than we could have had by the old *regime* under which men feared to become union men, or, having become such, confessed it blushingly, or lyingly denied it. I go further, and say that we have better masters, better employers, broader-gauged handlers of capital, because of this very proper consociation of the labor mind.

I have made this prefatory statement, in order to show that what I am about to say in criticism of a single feature of the trades union system is not presented with any prejudice against the unions as such, but with the broader and purer purpose of pointing out what seems to me a very grave mistake in the workings of these organizations; a mistake which I verily believe is counteracting many of the otherwise excellent effects of these unions. Perhaps I shall prove my point, and possibly I may not. In either event, what I say may raise a question in trades union assemblies which, though such question has doubtless frequently been handled at length and with ability by the membership, may be profitably rediscussed, and in the light of newer facts the damaging feature may at least be modified, if not abolished altogether.

It is a rule with all trades unions whose workings are familiar to me, that the number of apprentices in any shop, or mill, or factory, or other establishments in which skilled labor is employed, must be limited to a certain percentage of the number of journeymen or master workmen employed. This would not be so objectionable a feature were it not for the other fact that this percentage is always too small. I recognize the cause of the adoption of such a rule, nor do I go so far as to say, with many, that it has origin in a jealous fear that too many learners of a trade will overstock the particular guild and cheapen the skill in the particular line of mechanics or artisanship which any mentioned trades union may aim to protect. I know such argument is used, but it is not the basis of the rule or law limiting apprenticeship. It is claimed—and not without reason—that it is not for the best interest of the journeyman, but rather to his detriment as an ambitious worker, and one who would approach as near to perfection in his skill as possible, to have his time taken up and his operations—often of the most painstaking and delicate character, and requiring all the attention and dexterity he can command—interrupted by the frequent and annoying and time-wasting instructions which a shop full of apprentices would entail, to say nothing of the distracting effect of the oft-repeated questions of learners, a large share of whom must, in the natural order of things, be stupid, or garrulous, or curious beyond what is meet. This, not the fear of multiplying skill beyond the demand, is the argument against a larger percentage of apprentices in our mechanical rooms.

It would be well, in view of all this, for the convened artisans to consider this question in connection with another very important circumstance.

Boys are irrepressible. Girls are not altogether devoid of the same characteristic. When a boy or a girl fully makes up his or her mind to learn any special trade, he or she may be counted upon as sure to learn or try to learn that trade somehow. Moreover, added to this determination of the particular boy or girl, that of his or her parent or guardian may be depended upon to the same end. This demand, widened out, becomes what may be regarded as public sentiment, and as it is a principle in industrial, as well as other economies, that for every demand there must be a corresponding supply, it is very safe to reason that a supply will be provided to match this demand. Besides this, there is another element that comes to the reinforcement of this demand, to wit: antagonism. There is no power that exerts so strong a tendency in the production of certain results as opposition. The very effort on the part of our labor unions to prevent a given result, to wit: the diminishment of the number of rivals in a given line of mechanical or other skilled work, or of those who interrupt and distract master workmen in their mechanical manipulations, is often the cause of raising up those very rivals and distractors, and increasing the number beyond what it would be without such effort.

Now, all these forces—the determination of the youth, the pressure brought to bear by the parent or guardian, and the sympathizing and antagonism referred to—are effecting just the results we have named. And this brings me to my intimated conclusion, viz.: that the effect, indirect, of this barring out of apprentices or learners from our shops, mills and factories, has been the establishment, all over this land and all other civilized lands, of technical schools, polytechnic colleges and industrial establishments designed especially for the instruction of young men and young women in almost, if not quite, every trade, art, science and calling under the sun. There is now no vocation known to the world that may not be learned, not only theoretically but practically, without going near a shop, mill, factory or other place where manufactured articles are produced for the world's market.

And again, the instructors in these technical institutions are culled from the most skilled and most intelligent classes of men and women that can be found in the world's shops and mills and factories. They are picked up here, there, everywhere, and are the brightest men and women of their guild. To these are submitted the boys and girls that determine to acquire proficiency in an art or trade, but are denied the privilege by the mistaken trades unions, labor unions and other associations of the like, and expert workmen and workwomen are graduated from these institutions in larger numbers, in shorter time and with a higher grade of technical knowledge and skill than they could have acquired had they been taken into the shops and mills without any limit whatever. It may be said that they are not so practical; that they are taught rather the theory than the practice of what they learn. This is not true. By actual test, the young men and women from these schools are notably of a higher standard of expertness than are the shop-made journeymen or master workmen. It stands to reason that they should be such, not only by reason of the superiority of their instructors, but because of the more time and better attention given to the said instruction. Where instruction is the sole aim and object of men or women, it is reasonable to expect that they will impart instruction more thoroughly, in less time, and by constant study of the methods of imparting, do it in a more intelligent and systematic manner. The ordinary apprentice receives, at best, but the most meager instruction. He teaches himself more than he is taught. It does not pay the master mechanic to take his time from his work, often by the piece, to show a boy, possibly a stupid fellow, how to do his work. The boy possibly fears to interrupt the workman and receive a curse or a rebuff, or at least a merely partial instruction in what he wants to know. Four-fifths of the apprentices learn all they know from merely observing how others do their work, receiving no explanation of the principles of the work, or the tricks, or what is termed knack of the workman. It is only by long and constant practice, oftentimes, that he arrives at what a single word, or a few moments of explanation at most, would give him at once. But the paid instructor is where he is, simply to do this and nothing more; to make the steps toward proficiency as easy as possible and his pupil's acquirement as speedy as possible, and his attainment as thorough as possible. No wonder that our polytechnic school men are superior to our shop-made men in their mechanical achievement.

But there is still another very important consideration in favor of those taught in the technical schools. The moral standard is higher. To demonstrate this one needs only to visit the shops or mills where apprentices are being made into journeymen, and those technical institutions where learners are being made experts in any line of the mechanical arts. In the former the *morale* of the youth is seldom looked after at all. He is required to be present at a certain hour of the morning, and to remain at his post until the hour of quitting. Where he goes, or what he does before or after shop hours, is not questioned provided he is on hand when wanted, and keeps himself wide awake enough to jump at the call of any "jour" that may autocratically demand a service from him. During his first year or two he is the menial of the entire shop, and must do the bidding of any, bear the blame and abuse of any, bear the cuffs and kicks of any, and spend more than half his time doing odd jobs and chores which have no more relation to the calling he has come there to learn than has the drudgery of the kitchen in the domestic routine any relation to the embroidery of the boudoir. If he be a sensitive youth, his manliness is dwarfed and all his nobler instincts are smothered. If he be a hot-headed, impulsive fellow, he acquires more skill at low repartee, and more dexterity in shirking work, and more cunning in playing tricks on his elders than he does of proficiency in the trade he has set out to learn. He grows up a rough fellow at best, made so by the very nature of his surroundings as an apprentice. This is the general rule, to which, I admit, there are honorable but most surprising exceptions. On the other hand, the boy in the technical school is in no different case from the boy in the school where literary attainments are the object in view. He is treated as a learner among many learners. Degrading, servile labor, outside of the art he has come to learn, is not imposed upon him. He is not the acknowledged servant, or valet, or errand boy, or cad, of any. He hears no harsh language from angered superiors, and receives no buffetings from those who chance to be older or less unsophisticated than he. Boys and girls in the schools are treated like young men and women who have been inspired with a laudable ambition to become busy workers for their own support, useful and worthy members of society, and honored citizens of the commonwealth. The education and training are the very antipodes of the shop or the mill. One is almost culture, the other is almost brutalization. The contrasts are so strong that it does not require more than the statement of the facts to make us honor the one and deplore the other.

What then do our trades unions and our labor associations gain by limiting the number of apprentices that may be received into any one industrial establishment? They gain the condemnation of public opinion; an increased number of workers in the same guild; more expert and more intelligent workers for rivals and competitors; better men and women, morally and intellectually, and hence such as are more apt to displace them, other things being equal. Is it a gain for the members of our trades unions, or is it a loss? It is a gain to the world outside of the trades unions, and, by indirection, perhaps, a gain to the members of the unions.

On the whole, would it not be far better to abandon the apprentice system entirely, and thus compel all who would acquire a trade or skilled mechanical calling, to learn that vocation in our industrial and technical schools? Thus would all our mechanics become better workmen, and operators of every sort and kind become better skilled and more valuable to themselves and to those who employ them, both in a pecuniary and a moral sense. Is it not coming to this? The increase in the number of these schools, and the unpopularity attaching to the old apprentice methods, seem to indicate that this is what must come to pass.

